# What is the cause of P‑wave undersensing in this CRT-D device?

**DOI:** 10.1007/s12471-018-1131-3

**Published:** 2018-06-25

**Authors:** J. Schroemges, F. A. L. E. Bracke, B. M. van Gelder

**Affiliations:** Department of Electrophysiology, Catharina Hospital, Eindhoven, The Netherlands

## Answer

**Fig. 1:**
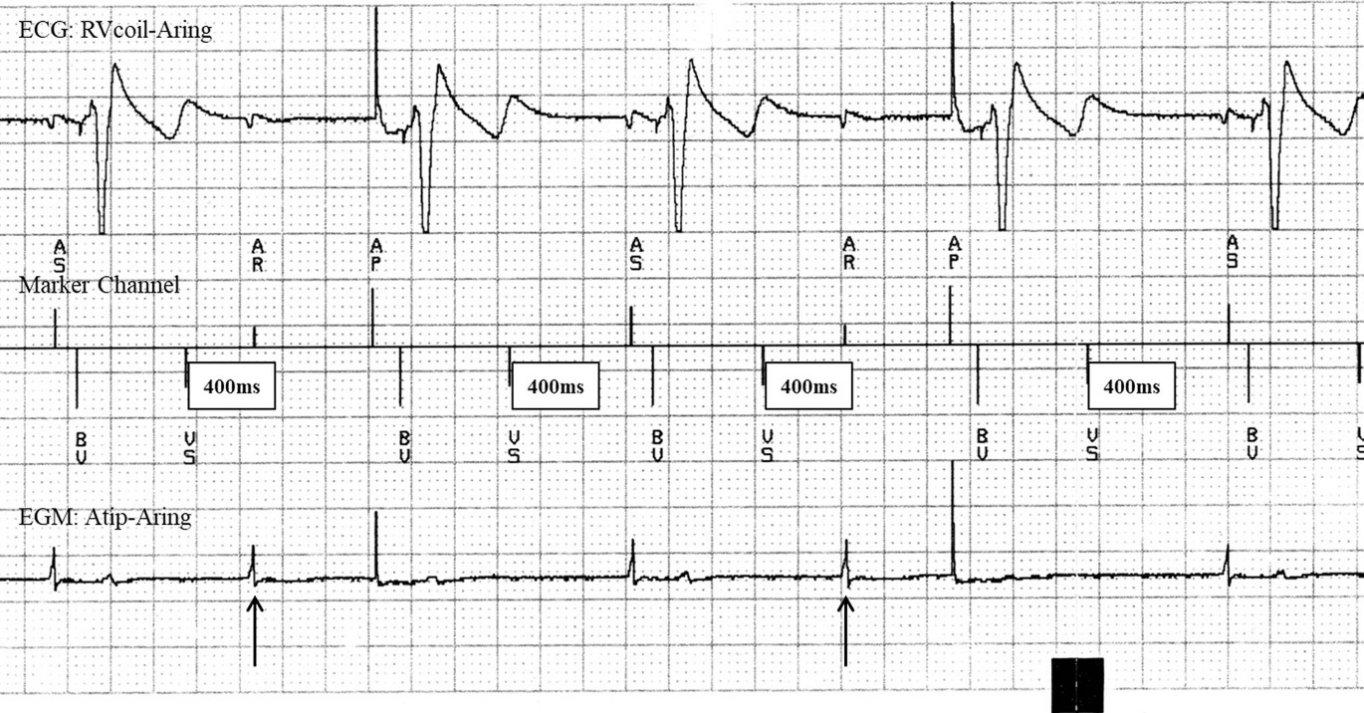
ECG and marker channel illustrate the mechanism of atrial undersensing. Ventricular sensing (*VS*) indicates T‑wave oversensing not preceded by a P wave, which initiates a 400 ms postventricular atrial refractory period (PVARP). The following P wave falls in this PVARP (indicated by *AR*) and is consequently not followed by ventricular pacing. This recording shows a 1:1 loss of pacing similar to the first two undersensing events in the figure in the question. In the second part of the figure in the question, 1:2 loss of pacing was observed, most likely related to small variations in the intrinsic atrial rate or changes in T‑wave amplitude, which can only be proven by marker channel and intracardiac EGM recordings

Undersensing of the intrinsic signal in cardiac devices has four possible causes. First, the intracardiac signal is inappropriate for sensing due to a low amplitude and/or slew rate; second, the intracardiac signal occurs in the refractory period of the device; third, reversion to a fixed rate pacing in case of continuous interference; and fourth, the amplitude of the electrogram is reduced by lead failure. Intracardiac recordings are helpful in differentiating these causes. After ventricular sensing or pacing the device starts a postventricular atrial refractory period (PVARP), which is programmable with a nominal setting of 320 ms. However, when ventricular sensing is not preceded by a P wave this ventricular beat is considered by the device to be a ventricular ectopic beat and the device switches to a PVARP of 400 ms, in order to prevent the initiation of pacemaker-mediated tachycardia, which might be evoked by ventricular ectopic beats with retrograde conduction. In Fig. 1, ventricular sensing is indicated in the marker channel by VS. From the ventricular EGM it is obvious that ventricular sensing is caused by T‑wave oversensing, which is not preceded by a P wave and is interpreted by the device to be a ventricular ectopic beat, resulting in an extended PVARP of 400 ms. By extending the PVARP to 400 ms the succeeding P wave falls in the extended PVARP (indicated by *AR *in Fig. 1) and is not followed by ventricular stimulation, resulting in electrocardiographic undersensing (Fig. 1). The problem was corrected by reducing ventricular sensitivity, which prevented T‑wave oversensing.

